# Battery pollutant leakage disrupts antioxidant ability and gut microbial homeostasis of chickens

**DOI:** 10.3389/fcimb.2025.1682969

**Published:** 2025-10-17

**Authors:** Xinxi Qin, Shuai Song, Guoqing Xiang, Shengmin Wang, Shengjun Luo, Caijuan Yang, Xiaohui Wen

**Affiliations:** ^1^ Institute of Animal Health, Guangdong Academy of Agricultural Sciences, Guangzhou, China; ^2^ Key Laboratory for Prevention and Control of Avian Influenza and Other Major Poultry Diseases, Ministry of Agriculture and Rural Affairs, Guangzhou, China; ^3^ Guangdong Province Key Laboratory of Livestock Disease Prevention, Guangzhou, China; ^4^ Department of Animal Science, College of Biology and Food, Shangqiu Normal University, Shangqiu, China; ^5^ Zhejiang Hisun Animal Healthcare Products Co., Ltd., Guangzhou, China; ^6^ National S&T Innovation Center for Modern Agricultural Industry, Guangzhou, China

**Keywords:** battery, pollutant, gut microbiota, diversity, chicken

## Abstract

Over the past few decades, battery industry and electronic equipment have undergone explosive growth, but the heavy metal waste generated has led to significant global ecological and public health challenges. Currently, increasing evidences have confirmed the detrimental effects of heavy metal exposure on animal reproduction, immunity, and metabolism. However, research focused on the impacts of battery leakage on the gut microbiota remain scarce. Thus, this study aims to investigate the detrimental effects of battery on gut microbiota in chickens. Results revealed that battery exposure can lead to a significant increase in spleen index and a significant decrease in thymus index in chickens. Furthermore, battery exposure can significantly increase serum ALT, AST and MDA levels, and while concurrently reducing levels of GSH-Px and SOD. Battery exposure also cause a significant reduction in the gut microbial alpha diversity, accompanied by significant alterations in taxonomic composition. Bacterial taxonomic analysis indicated that the relative abundances of 1 phyla and 4 genera increased dramatically, while the relative abundance of 3 phylum and 115 genera decreased significantly during battery exposure. In conclusion, this study suggests that battery exposure leads to gut microbial dysbiosis and affect antioxidant ability in chickens. The significant alterations of gut microbiota may represent one of the mechanisms through which battery exerts its intestinal and renal toxicity. Given the context of battery pollutant leakage and inadequate recycling supervision, this study contributes to providing impetus for environmental protection agencies and organizations worldwide to enhance the recycling of battery waste.

## Introduction

Batteries, including lithium-ion, zinc-manganese, and nickel-chromium types, serve as the primary power sources for various mobile electronic devices such as smartphones and computers (Xiao et al., 2021). Moreover, they are also recognized as highly eco-toxic contaminants. Over the past few decades, the widespread use and rapid advancement of mobile electronic devices have led to explosive growth in the battery industry. According to statistics, the global lithium-ion battery market size reached approximately USD 29.86 billion in 2017, with continued gradual growth. China is one of the major battery consumers, with over 25 billion waste lithium-ion batteries generated in 2020, amounting to nearly 500,000 tons of production. Unfortunately, a substantial portion of these waste batteries cannot be effectively recycled, resulting in their release into the ecological environment and contributing to severe global environmental issues (Xie et al., 2017; Gottesfeld et al., 2018). Surveys indicate that the growth rate of battery waste was as high as 8% in 2018, with projections suggesting a further increase of 18% to 30% by 2030. Although some waste battery recycling technologies have emerged, the management and control of waste batteries remain severely constrained due to inadequate institutional support for recycling facilities (Zhu et al., 2017; Urrutia-Goyes et al., 2018). Consequently, most batteries are ineffectively managed, often disposed of through deep burial, composting, or incineration. Battery waste predominantly contains heavy metals, organic solvents, and plastic fragments, which can accumulate in water and soil, leading to significant biosafety and ecological concerns. Furthermore, battery waste is resistant to rapid degradation and may bioaccumulate in aquatic organisms, insects, and plants, thereby posing risks to food safety and animal health through the food chain (Debrah et al., 2024). Despite the rapid growth of the new battery industry, the potential risks that battery waste poses to animal health remain inadequately addressed.

The intestine serves as the primary channel for food intake and harbors approximately 100 trillion microorganisms, including bacteria, fungi, and viruses (Biagi et al., 2017; Tong et al., 2022). These microorganisms, namely the gut microbiota, play a crucial role in host health and various physiological functions by establishing a complex symbiotic relationship with the host (Huang et al., 2020; Passos and Chaturvedi, 2021). Research has demonstrated that the gut microbiota can metabolize carbohydrates from food to produce short-chain fatty acids (SCFAs), vitamins, and antimicrobial peptides, which positively regulate resistance to pathogenic bacterial infections and enhance growth and development (Mehmood et al., 2023; Nogal et al., 2021). Furthermore, the gut microbiota has also been shown to be involved in the maturation of the immune system, the maintenance of intestinal barrier function, and bone development (Jabeen et al., 2023; Yan et al., 2022; Wang et al., 2023). Numerous studies have indicated that the stability of the gut microbiota is closely linked to the maintenance of host health and various complex physiological functions (Zhang et al., 2023b). Conversely, the gut microbial dysbiosis can adversely affect host health. Typically, the gut microbiota is influenced by a variety of internal and external factors, resulting in dynamic changes (Celorrio et al., 2021; Chen et al., 2025a). Notably, external factors such as antibiotics, heavy metals, and microplastics can significantly impact the composition and structure of the gut microbiota, potentially inducing gut microbial dysbiosis (Jubair et al., 2018; Liu et al., 2022). This imbalance not only disrupts intestinal function but also extends its detrimental effects beyond the gastrointestinal system, leading to systemic consequences (Jiang et al., 2019). Studies have shown that the gut microbial dysbiosis can increase intestinal permeability, subsequently contributing to the development of diseases such as enteritis, diarrhea, and colitis (Shen et al., 2022; Wang et al., 2024; Xie et al., 2024). Recent studies focusing on gut microbial dysbiosis have also revealed its important role in diseases such as diabetes, Parkinson’s syndrome, allergies, and obesity. Consequently, any factor that disrupts the homeostasis of gut microbiota warrants special attention (Wu et al., 2020).

Chickens have increasingly become a significant source of protein for humans, attributed to their rapid growth rate and nutritional value (Li et al., 2025; Wu et al., 2024). According to statistics, the total global chicken production in 2024 is 103.046 million tons, with a market size estimated at approximately US$217.75 billion. In the same year, China’s chicken production is approximately 15 million tons, representing 14.56% of the global total and ranking second worldwide. Furthermore, it is predicted that China’s per capita chicken consumption will reach 15.2 kilograms in 2025, while Hong Kong’s per capita consumption is projected to be as high as 55.52 kilograms. As a major producer and consumer of chickens, the production and health of chickens in China are closely linked to the lives of residents. Previous research indicated that exposure to waste batteries can significantly impact the liver and kidneys of mice (Liao et al., 2022). Similarly, Wang et al. (2022) have demonstrated the toxic effects of extracts from waste batteries on zebrafish. However, there is a paucity of studies investigating the effects of used battery pollutant leakage on chickens health. Therefore, this study aims to explore the impacts of waste battery exposure on the gut microbiota and antioxidant ability of chickens.

## Materials and methods

### Experimental design and sample acquisition

In this study, 40 healthy AA chickens of similar body weight were procured from a commercial hatchery. The chickens were housed collectively for three days to minimize the impact of stress reactions on the experiment. Subsequently, they were randomly divided into two groups: control group (CON) and battery exposure group (EXE), each consisting of 20 chickens. The chickens were maintained in a standard environment with controlled temperature (from 33°C~35°C during the first week, gradually decreased to 29°C at the end of the second week), humidity (60%~65%), and lighting (23 h/1h light/dark cycle) to ensure optimal growth conditions. Adequate diet and drinking water were provided to all chickens throughout the duration of the experiment. Notably, battery waste was added to the water (230 mg/L) of the experimental group to induce battery poisoning. In addition, the main components of battery waste include cobalt, nickel, manganese, iron, copper, calcium, sodium and zinc. During the experiment, feed intake, average daily weight gain, and changes in body weight were recorded to compare the effects of battery exposure on the growth performance of chickens. The experimental period was based on previous studies (Liao et al., 2022). After 28 days, all chickens were euthanize using pentobarbital (25 mg/kg) (Hou et al., 2025). The serum, spleen, bursa of Fabricius, and thymus were collected to assess changes in immune organ indices. Concurrently, the cecal contents of the chickens were immediately collected and snap-frozen in liquid nitrogen for amplicon sequencing.

### Biochemical assays

Serum biochemical indicators including ALT, AST, and T-AOC, GSH-Px, CAT, SOD and MDA were measured using commercial kits in accordance with established methodologies from previous studies (Liao et al., 2022).

### DNA extraction and amplicon sequencing

Amplicon sequencing of the gut microbiota was conducted following previous established protocols (Hou et al., 2025; Peng et al., 2024). Briefly, DNA from cecal contents was extracted in accordance with the kit instructions. After assessing the DNA quality, qualified DNA was amplified using primers (338F: ACTCCTACGGGAGGCAGCA and 806R: GGACTACHVGGGTWTCTAAT). The PCR reaction system and conditions were established based on prior studies (Hou et al., 2025; Peng et al., 2024). Following PCR, the amplified products were purified, quality assessed, and quantified to prepare sequencing libraries. The constructed libraries underwent an initial quality check, and only those with concentrations exceeding 2 nM were sequenced using the Illumina NovaSeq 6000 platform. Given that the raw data from high-throughput sequencing contained unqualified sequences, a series of processing steps, including quality filtering and DADA2 denoising, were implemented to obtain valid sequences. Briefly, quality screening and primer elimination of original data were conducted to achieve clean reads devoid of defective, short, or mismatched sequences utilizing Trimmomatic (v0.33) and Cutadapt software (1.9.1). The resulting clean reads were then spliced and subjected to a secondary filter based on the length of the spliced sequences utilizing Usearch software (v10). Subsequently, chimera sequences in the raw data were identified and removed using UCHIME software (v4.2) to yield effective reads. The valid sequences from each sample were clustered into OTUs based on 97% sequence similarity. Additionally, the alpha diversity index of the gut microbiota was calculated based on the number of OTUs in each sample. Simultaneously, the PCoA diagram was also generated to visualize the structure of the gut microbiota. The data were presented as Mean ± standard deviation (SD). Metastats and LEfSe analyses were employed to identify differential taxa associated with battery exposure, using a significance threshold of P < 0.05 or LDA > 4.

## Results

### Effects of battery exposure on growth performance and organ indices in chickens

**Figure 1:**
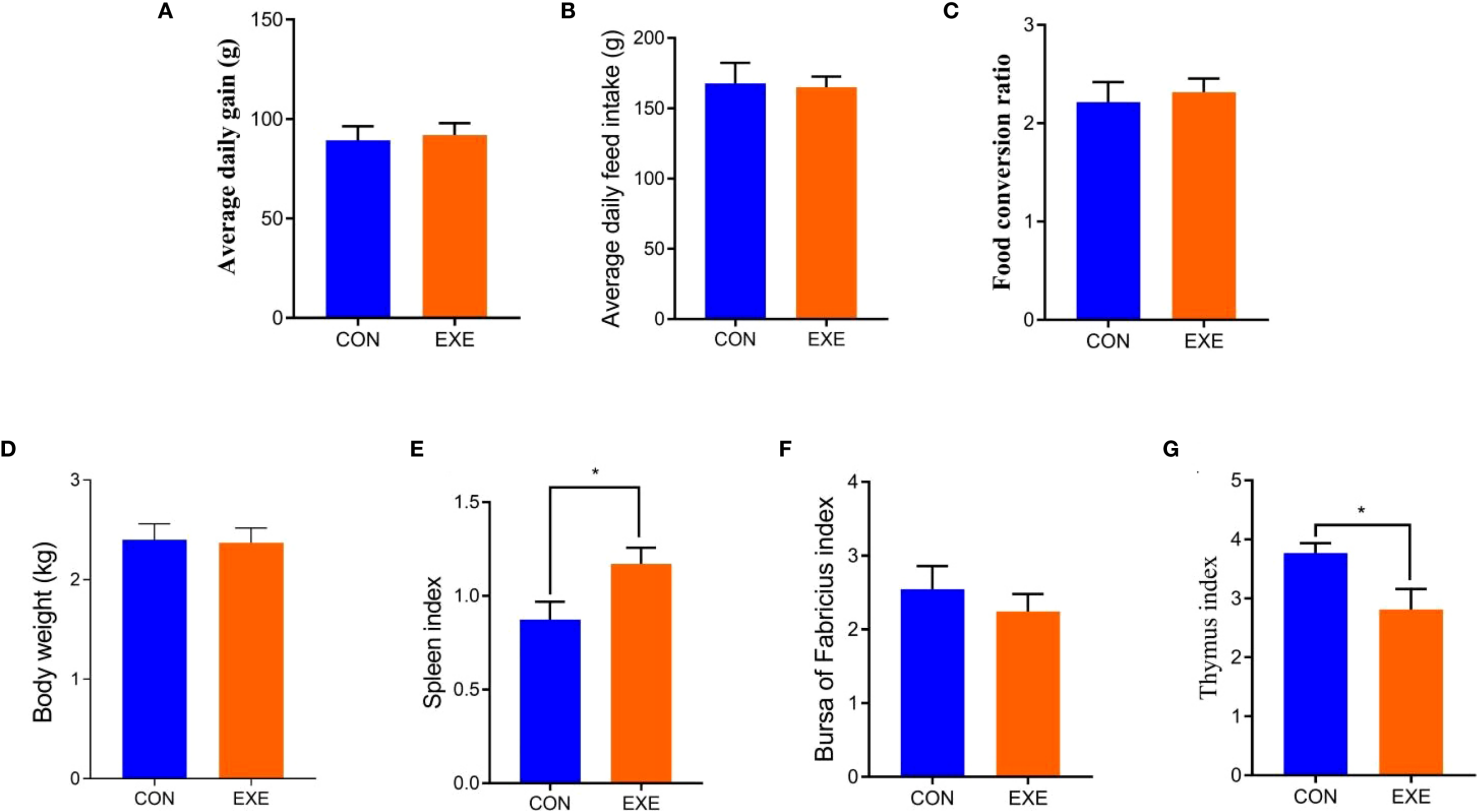
Effects of battery exposure on growth performance-related parameters in broiler chickens. **(A)** average daily gain; **(B)** average daily feed intake; **(C)** food conversion ratio; **(D)** Body weight; **(E)** spleen index; **(F)** bursa of fabricius index, **(G)** thymus index. The data was expressed as mean ± SD. *P < 0.05.

Body weight changes and organ indices of chicken in each group are present in **Figure 1**. Results revealed that battery exposure had no effect on the body weight gain, average daily weight gain, average daily feed intake and food conversion ratio of the chickens (**Figures 1A-D**). Organ index analysis revealed that exposure to batteries did not affect the bursa index of broiler chickens, but it significantly increased the spleen index and markedly decreased the thymus index (P < 0.05) (**Figures 1E-G**).

### Effects of battery exposure on serum biochemical indices in chickens

**Figure 2:**
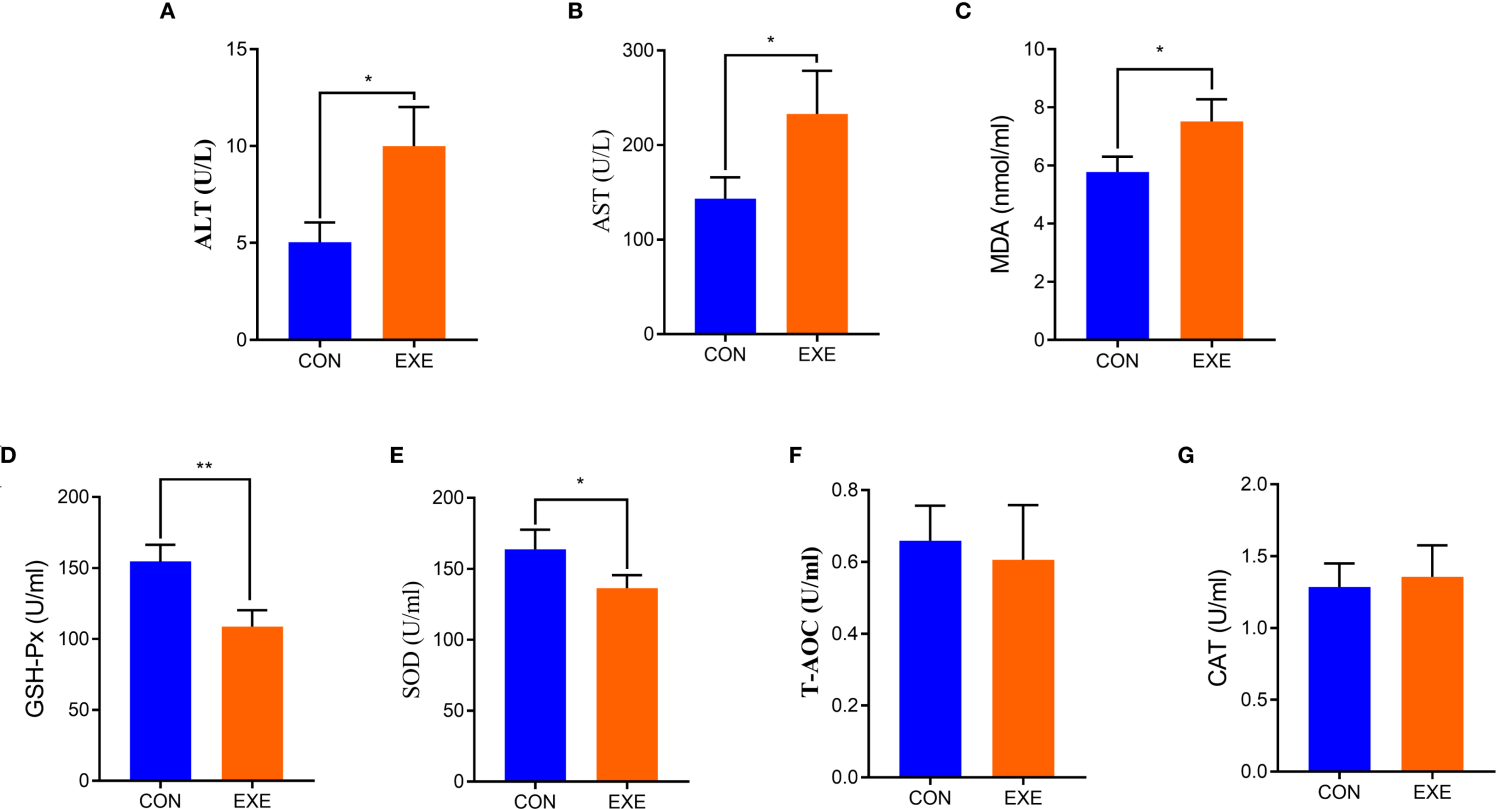
Effects of battery exposure on serum biochemical parameters in broiler chickens. **(A)** ALT; **(B)** AST; **(C)** MDA; **(D)** GSH-Px; **(E)** SOD; **(F)** T-AOC, **(G)** CAT. The data was expressed as mean ± SD. *P < 0.05, **P < 0.01.

Serum biochemical analysis showed that the levels of ALT and AST in the EXE were higher than those in the CON (P < 0.05) (**Figures 2A, B**). Additionally, we also observed that battery exposure dramatically reduced the antioxidant ability of chicken, characterized by increased levels of MDA and decreased levels of SOD, and GSH-Px (P < 0.05 or P < 0.01) (**Figures 2C-E**). Notably, battery exposure had no significant effect on the levels of CAT and T-AOC (**Figures 2F, G**).

### Sequence analysis

**Figure 3:**
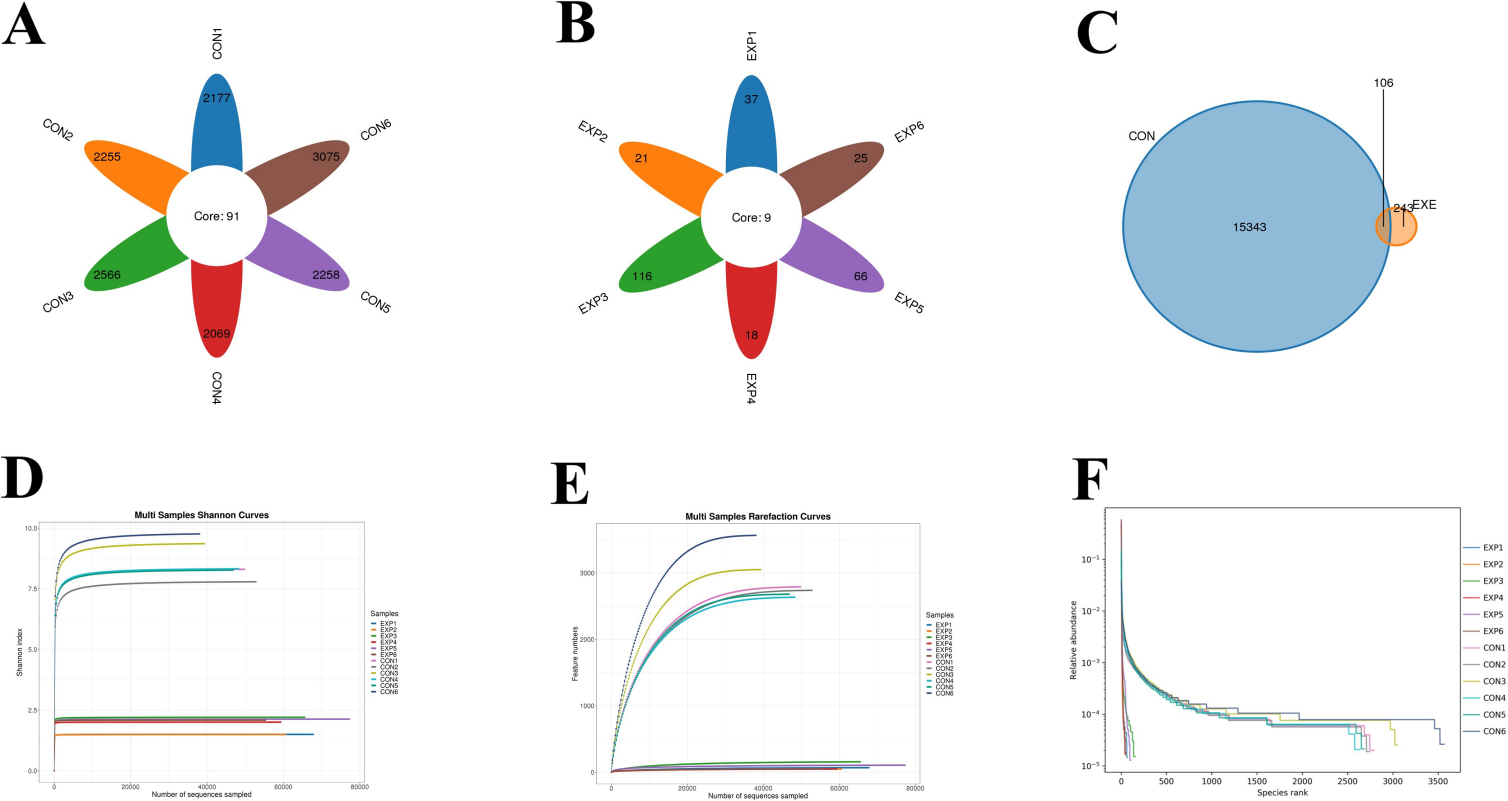
Effects of battery exposure on the diversity of gut microbiota. **(A-C)** The number of OTUs generated from valid sequences. **(D-F)** Feasibility analysis of the results obtained from gut microbiota sequencing.

In this study, microbiome sequencing was performed on samples collected from both the CON and EXE groups, generating a total of 1,381,564 (CON=853,910, EXE=527,654) raw sequences. After removing questionable sequences, 697,050 (CON=309,604, EXE=387,446) valid sequences were identified, yielding a qualification rate of over 50% (**Table 1**). These valid sequences were subsequently clustered into 15,692 OTUs, with 15,449 and 349 OTUs identified in the CON and EXE, respectively (**Figures 3A, B**). Additionally, 106 OTUs were shared between both groups, accounting for 0.67% of the total OTUs (**Figure 3C**). The individual OTU counts in the CON and EXE were 15,343 and 243, respectively, which represented 98.45% and 2.22% of their respective OTU totals. Importantly, the rarefaction curve analysis, which was employed to assess sequencing depth, revealed that the curves reached saturation, indicating that the sequencing depth was adequate (**Figures 3D, E**).

**Table 1:** Sequence analysis of gut bacterial community sequencing in the CON and EXE.

Sample ID	Raw reads	Clean reads	Denoised reads	Merged reads	Non-chimeric reads
CON1	150003	131885	130418	113029	54852
CON2	136639	120852	119557	102906	57828
CON3	140644	125025	123316	100598	45879
CON4	146768	128312	126926	108360	53087
CON5	138159	121980	120502	102995	51873
CON6	141697	126665	124823	100565	46085
EXP1	82909	74667	74651	74517	68022
EXP2	75298	67874	67850	67683	60691
EXP3	89830	80276	80236	79918	65850
EXP4	94887	85575	85528	85292	59653
EXP5	91989	82144	82138	81412	77571
EXP6	92741	83073	83020	82928	55659

### Battery exposure changed the gut microbial diversity

**Figure 4:**
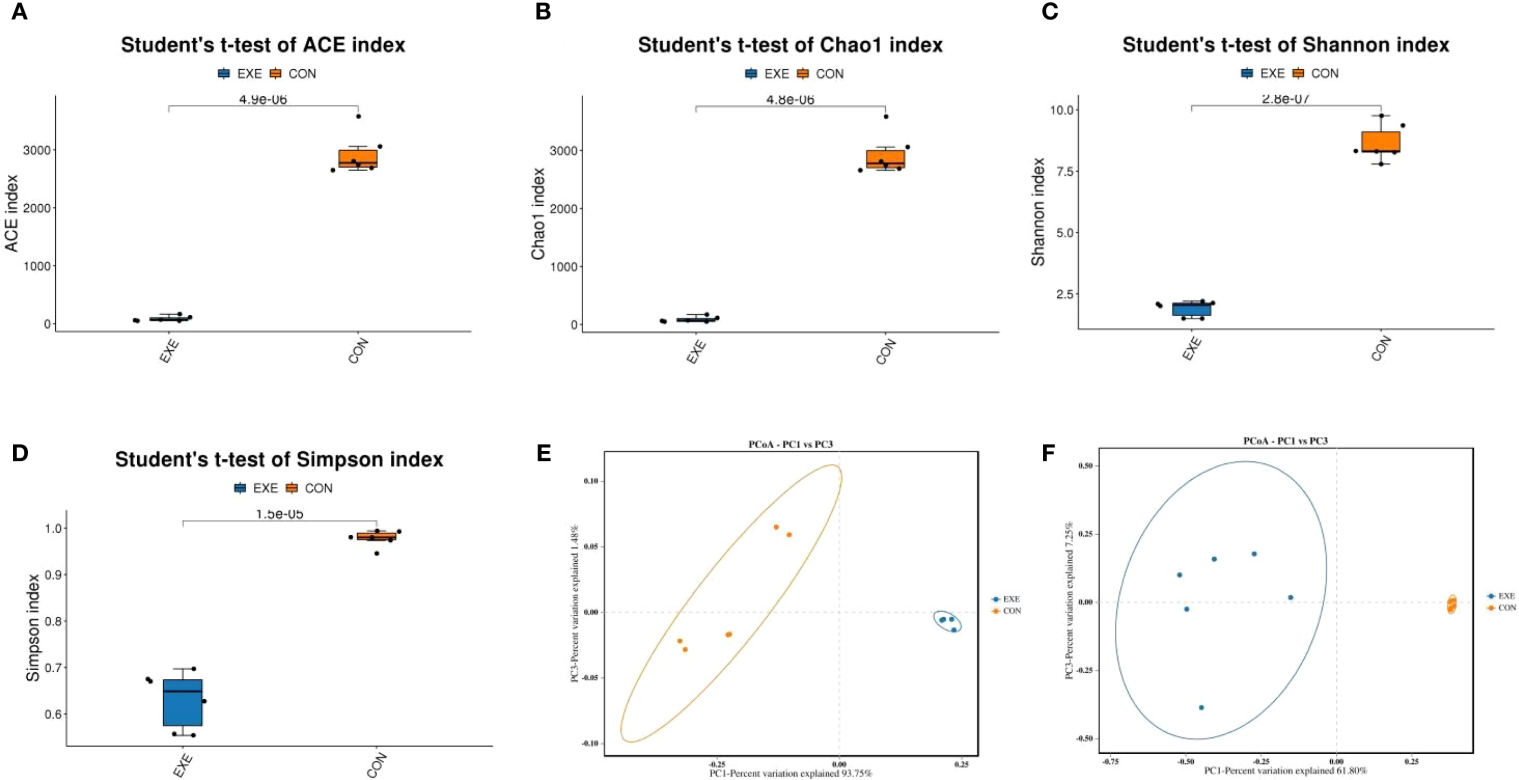
Effects of battery exposure on the diversity of gut microbiota in broiler chickens. **(A-D)** The ACE, Chao1, Shannon and Simpson indices were used for comparing the diversity and abundance. **(E, F)** PCoA scatter plots were generated to visualize the differences in the structure of gut microbiota. The data was expressed as mean ± SD.

To further compare the differences in gut microbial diversity between the CON and EXE, we calculated the alpha diversity index based on the abundance of OTUs in each sample. The results indicated that the average values of the ACE index were 2920.75 for the CON and 84.52 for the EXE, while the average Chao1 index values were 2924.76 and 86.08, respectively. Furthermore, the average values of Shannon index for the CON and EXE was 8.63 and 1.90, while and the average values of Simpson index was 0.97 and 0.63, respectively. Statistical comparisons revealed that battery exposure significantly reduced the gut microbial Chao1 (2924.76 ± 144.26 vs. 86.08 ± 19.39, P < 0.001) and ACE (2920.75 ± 144.15 vs. 84.52 ± 18.59, P < 0.001), Simpson (9.07± .0.72 vs. 0.63±0.025, P < 0.001) and Shannon (8.63±0.30 vs. 1.90 ± 0.13, P < 0.001) indices (**Figures 4A-D**). These findings suggested that battery exposure significantly decreased the abundance and diversity of gut microbiota. Additionally, beta diversity analysis based on the PCoA scatter plot demonstrated that samples from the CON and EXE were significantly separated, indicating that battery exposure significantly alter the structure of the gut microbiota (**Figures 4E, F**).

### Battery exposure changed the gut microbial composition

**Figure 5:**
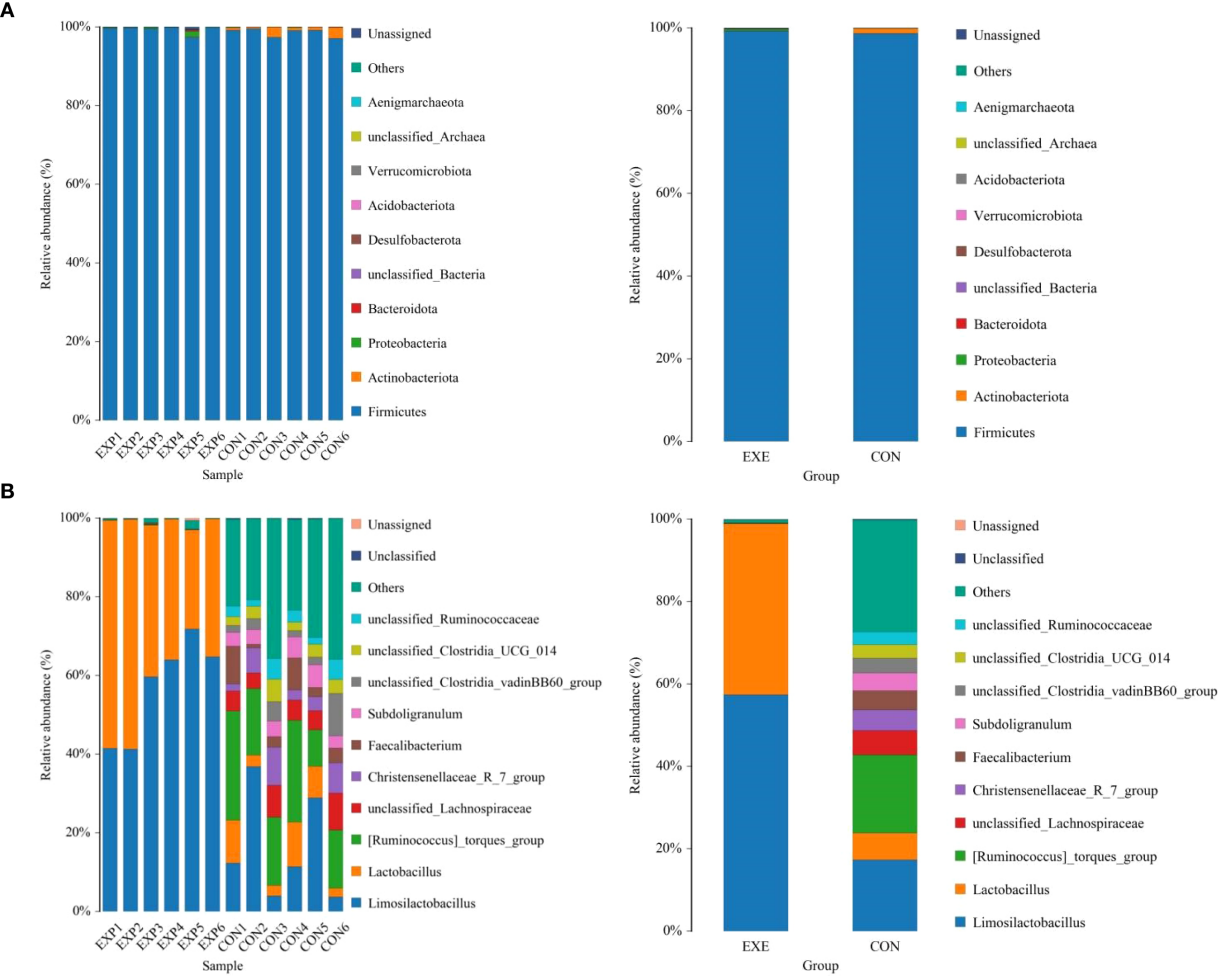
Effects of battery leakage on gut microbiota of broiler chickens. **(A)** phyla leval. **(B)** genus level. Each bar represents the average relative abundance of each bacterial taxon within a group.

**Figure 6:**
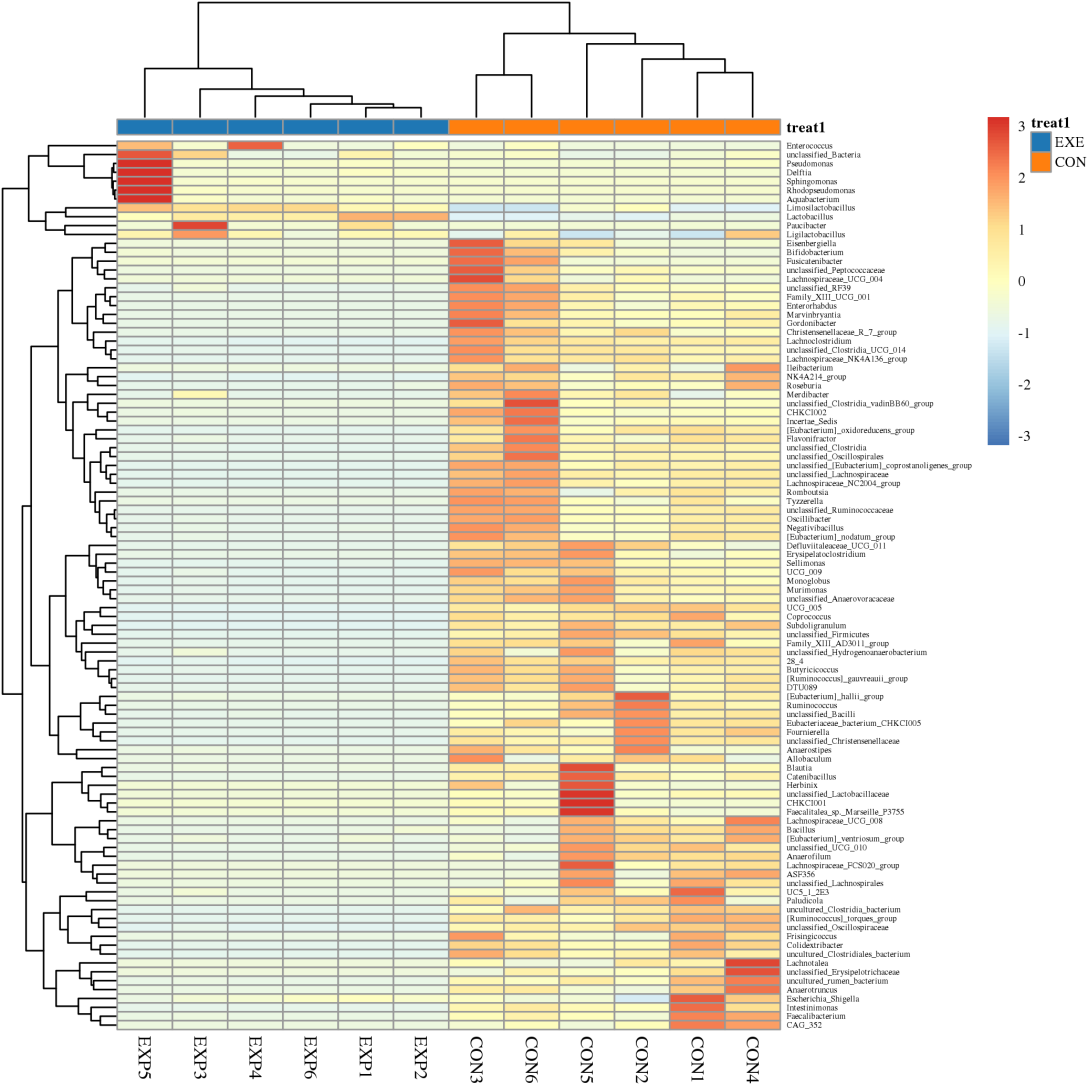
Cluster heat map analysis of gut microbiota. The relative abundance of gut microbiota is positively correlated with color depth. The values in the heat map represent the square-root-transformed relative abundance of each bacterial genus. The intensity of color in the heat map corresponds to the square-root-transformed values of the bacterial genera, with the legend located in the upper right corner of the figure.

At the phylum level, *Firmicutes* (98.68%), *Actinobacteriota* (1.186%), *Proteobacteria* (0.057%) and *Bacteroidota* (0.037%) were identified as the most preponderant in the CON. As for the EXE, *Firmicutes* (99.27%), *Proteobacteria* (0.40%), *unclassified_Bacteria* (0.064%) and *Bacteroidota* (0.059%) constituted a significant proportion (**Figure 5A**). Other phyla such as *Verrucomicrobiota* (0.0047%, 0.0012%), *Acidobacteriota* (0.0025%, 0.0033%), *unclassified_Archaea* (0.00%, 0.0031%) and *Aenigmarchaeota* (0.00%, 0.0038%) in CON and EXE were recognized in low ratios. At the genus level, the *Ruminococcus_torques_group* (18.93%, 0.047%), *Limosilactobacillus* (17.30%, 57.39%) and *Lactobacillus* (6.54%, 41.50%) were abundantly present in the CON and EXE (**Figure 5B**). The abundance of more bacterial genera in the CON and EXE can also be displayed through cluster heat maps (**Figure 6**).

**Figure 7:**
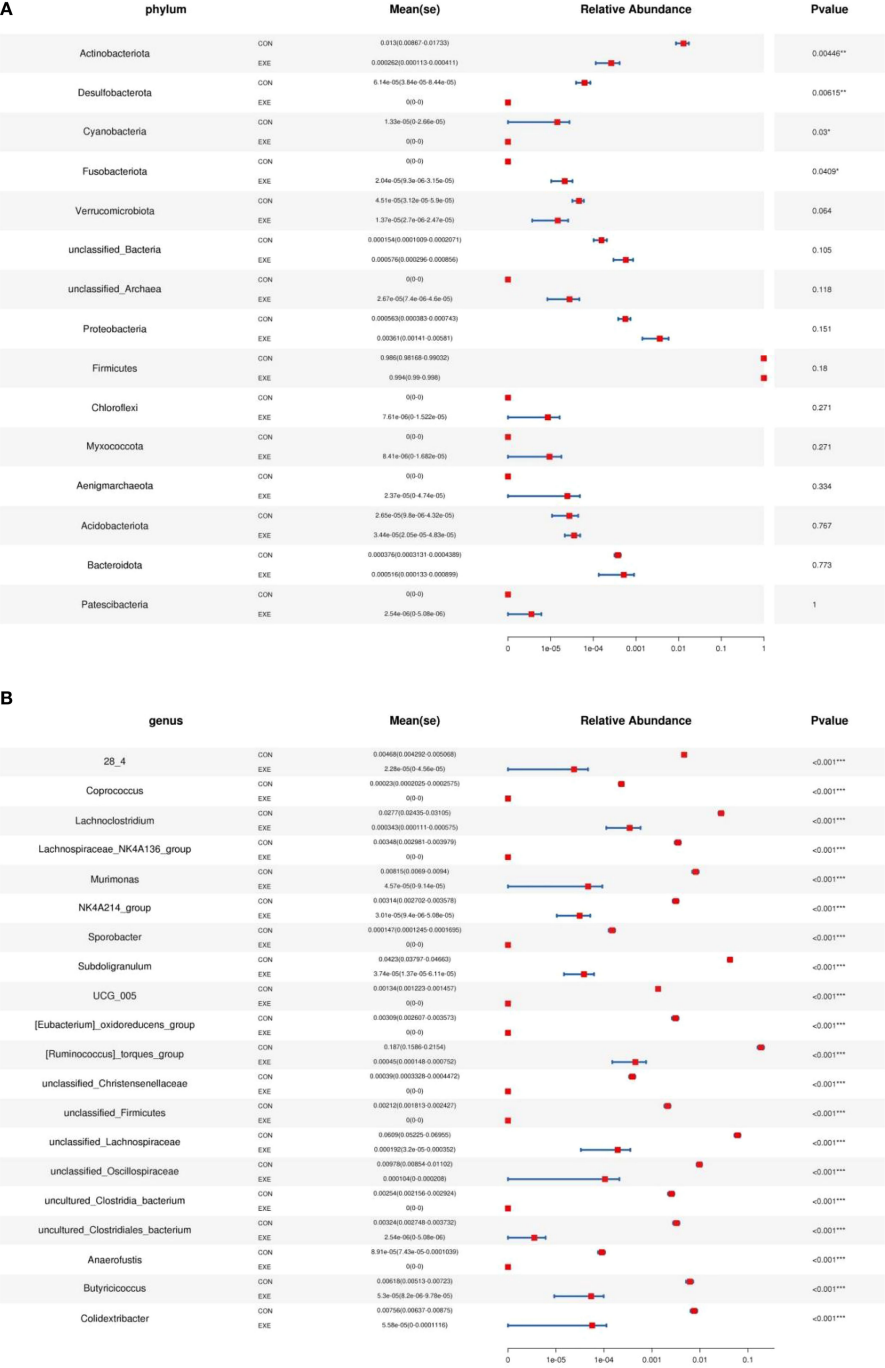
Metastats analysis was used to identify differential taxa at the phylum **(A)** and genus **(B)** levels. The data was expressed as mean ± SD. **P<0.01, ***P<0.001.

**Figure 8:**
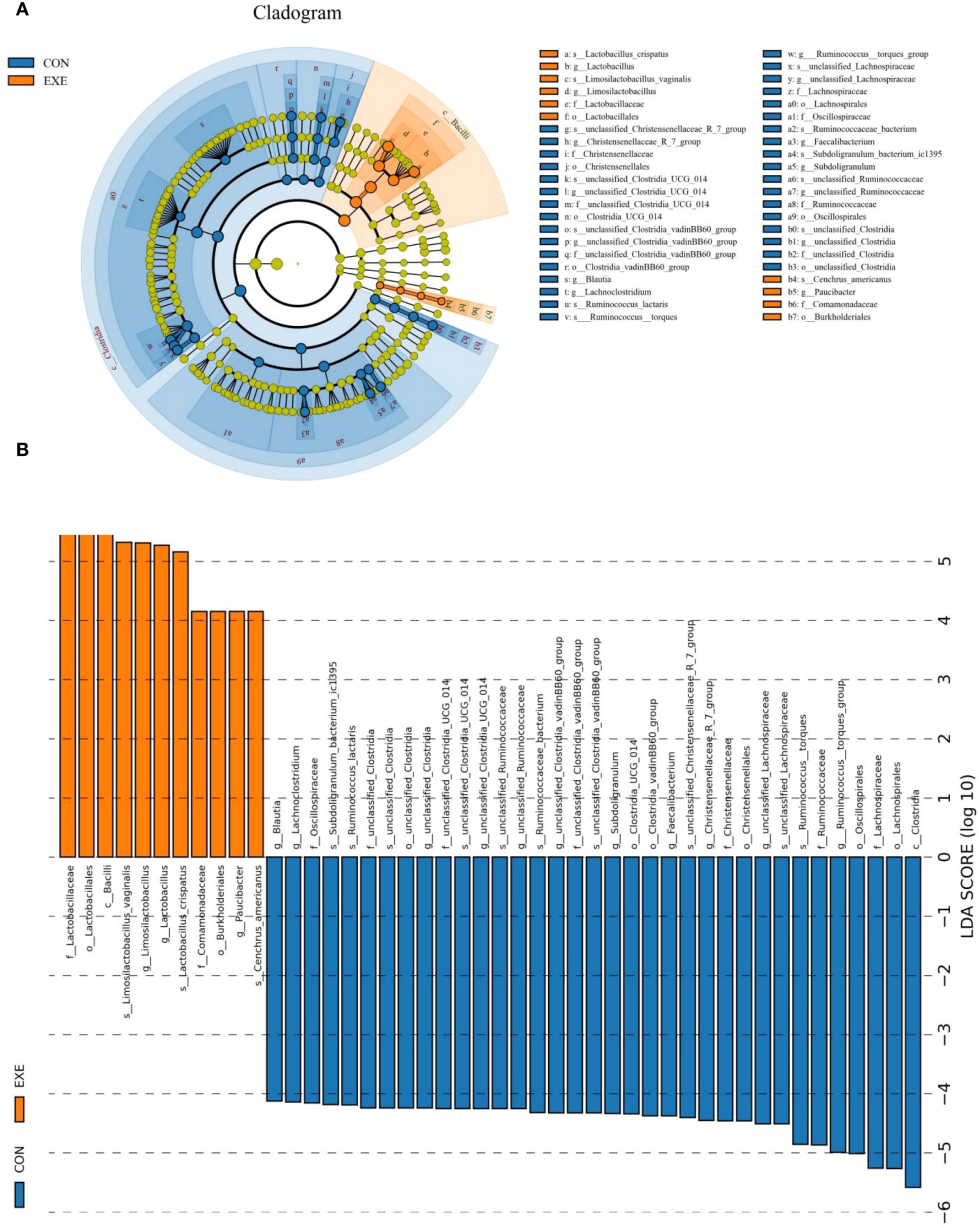
The differential taxa at phyla and genus levels were visualized by LEfSe analysis. **(A)** Evolutionary relationships of different species at different taxonomic levels. **(B)** LDA values ≥ 4 were set as the identification criteria for differential bacteria.

Metastats and LEfSe analysis was used for identifying differential taxa associated with battery exposure. At the phylum level, *Fusobacteriota* was dramatically more preponderant in the EXE than in the CON, while the *Actinobacteriota*, *Desulfobacterota* and *Cyanobacteria* were lower (**Figure 7A**). Moreover, a total of 119 genera were found to be dramatically different between EXE and CON. Among them, the proportions of 4 bacterial genera (*Mycoplasma*, *uncultured_Acidobacteria_bacteriu m*, *Limosilactobacillus* and *Lactobacillus*) dramatically increased, while the relative richness of 115 bacterial genera (*Coprococcus*, *Lachnoclostridium*, *Lachnospiraceae_NK4A136_group*, *Murimonas*, *Sporobacter*, *Subdoligranulum*, *Ruminococcus_torques_group*, *Anaerofustis*, *Butyricicoccus*, *Colidextribacter*, *Lachnospiraceae_NC2004_group*, *Monoglobus*, *Ruminococcus_gauvreauii_group*, *Fournierella*, *Sellimonas*, *Oscillibacter*, *Candidatus_Soleaferrea*, *Alistipes*, *Flavonifractor*, *Defluviitaleaceae_UCG_011*, *Intestinimonas*, *Negativibacillus*, *Christensenellaceae_R_7_group*, *Marvinbryantia*, *Ruminococcus*, *Roseburia*, *Catenibacillus*, *Romboutsia*, *Erysipelatoclostridium*, *Lactonifactor*, *Anaerofilum*, *Enterorhabdus*, *Gordonibacter*, *Tyzzerella*, *Frisingicoccus*, *Anaerostipes*, *Faecalibacterium*, *Incertae_Sedis*, *Paludicola*, *Bacillus*, *Anaerotruncus*, *Lachnospiraceae_UCG_008*, *Allobaculum*, *Blautia*, *Ileibacterium*, *Lachnospiraceae_UCG_010*, *Lachnospiraceae_UCG_006*, *Lachnospiraceae_FCS020_group*, *Acetitomaculum*, *Bacillaceae_bacterium_BM62*, *Faecalibaculum*, *Lachnotalea*, *Bifidobacterium*, etc.) significantly decrease during battery exposure (**Figure 7B**). Notably, battery exposure even resulted in 68 bacterial genera (*Coprococcus*, *Lachnospiraceae_NK4A136_group*, *Sporobacter*, *Anaerofustis*, *Lachnospiraceae_NC2004_group*, *Defluviitaleaceae_UCG_011*, *Intestinimonas*, *Catenibacillus*, *Romboutsia*, *Lactonifactor*, *Anaerofilum*, *Enterorhabdus*, *Gordonibacter*, *Tyzzerella*, *Frisingicoccus*, *Anaerotruncus* and *Lachnospiraceae_UCG_008*, etc.) cannot be recognized in the gut microbiota. Moreover, LEfSe analysis indicated that the CON was significantly enriched for *Ruminococcus:torques_group*, *unclassified_Lachnospiraceae*, *Faecalibacterium*, *Subdoligranulum*, *unclassified_Clostridia_vadinBB60_group*, *unclassified_Clostridia*, *unclassified_Ruminococcaceae*, *unclassified_Clostridia_UCG_014* and *Lachnoclostridium*, while the EXE indicated a significantly higher abundances of *Limosilactobacillus*, *Lactobacillus* and *Paucibacter* (**Figure 8**).

### Correlation network analysis

**Figure 9:**
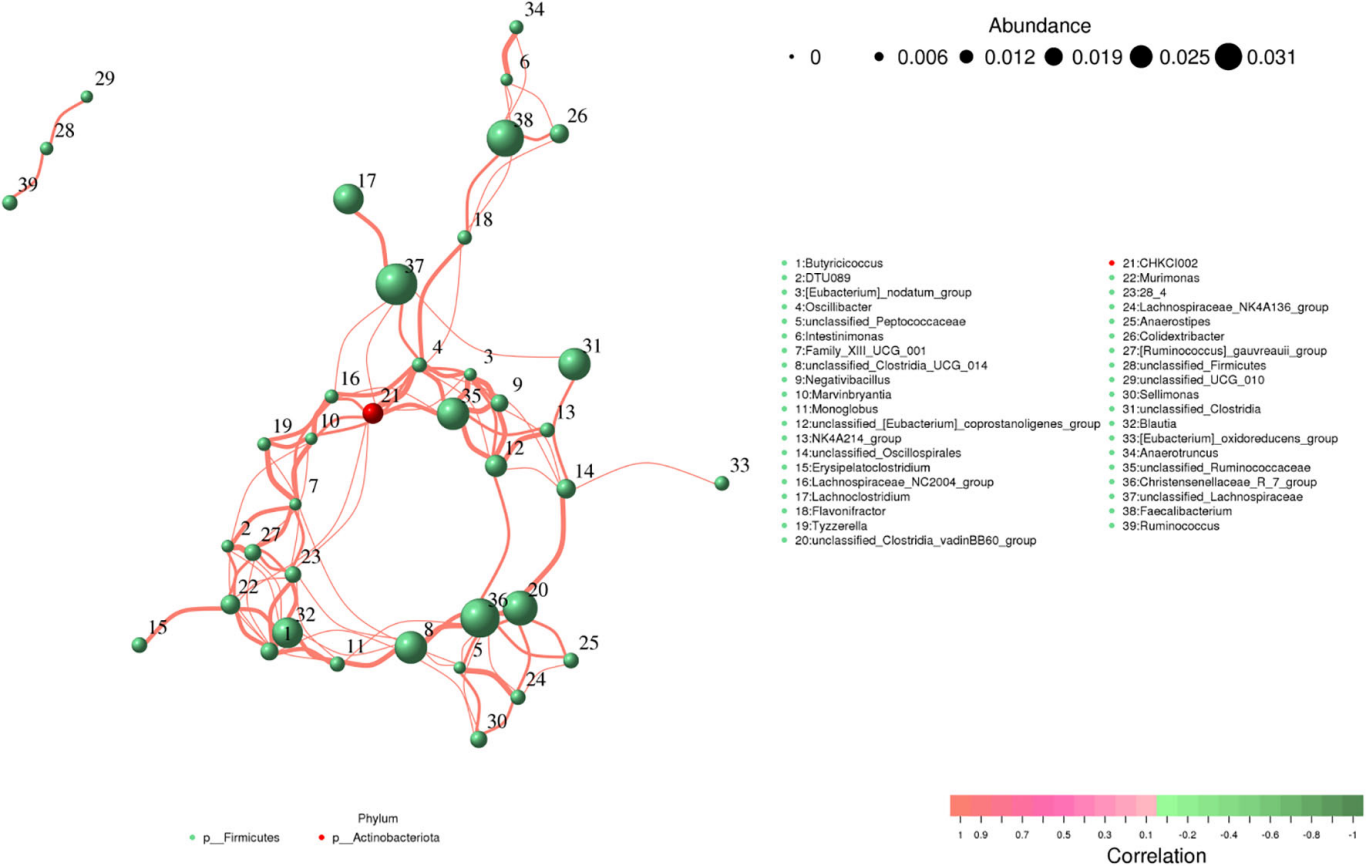
Correlation analysis of intestinal flora. The correlation between bacteria is shown by line segments. The thickness of the lines indicates the strength of the correlations, where orange lines represent positive correlations.

*Butyricicoccus* showed a positive association with *Blautia* (0.99), *Murimonas* (0.96) and *unclassified_Clostridia_UCG_014* (0.94). *Intestinimonas* was positively associated with *Anaerotruncus* (0.99), *Faecalibacterium* (0.95), *Colidextribacter* (0.94) and *Flavonifractor* (0.94) (**Figure 9**). *Monoglobus* was positively associated with *unclassified_Clostridia_UCG_014* (0.97), *Blautia* (0.97), *Butyricicoccus* (0.97), *Murimonas* (0.95), *28_4* (0.95) and *Christensenellaceae_R_7_group* (0.94). *Lachnospiraceae_NC2004_group* was positively associated with *Oscillibacter* (0.97), *unclassified_Ruminococcaceae* (0.94), *CHKCI002* (0.96), and *unclassified_Lachnospiraceae* (0.95). *Flavonifractor* was positively closely related to *Oscillibacter* (0.96), *Faecalibacterium* (0.95), *CHKCI002* (0.94) and *Colidextribacter* (0.94).

### Battery exposure changed the intestinal function

**Figure 10:**
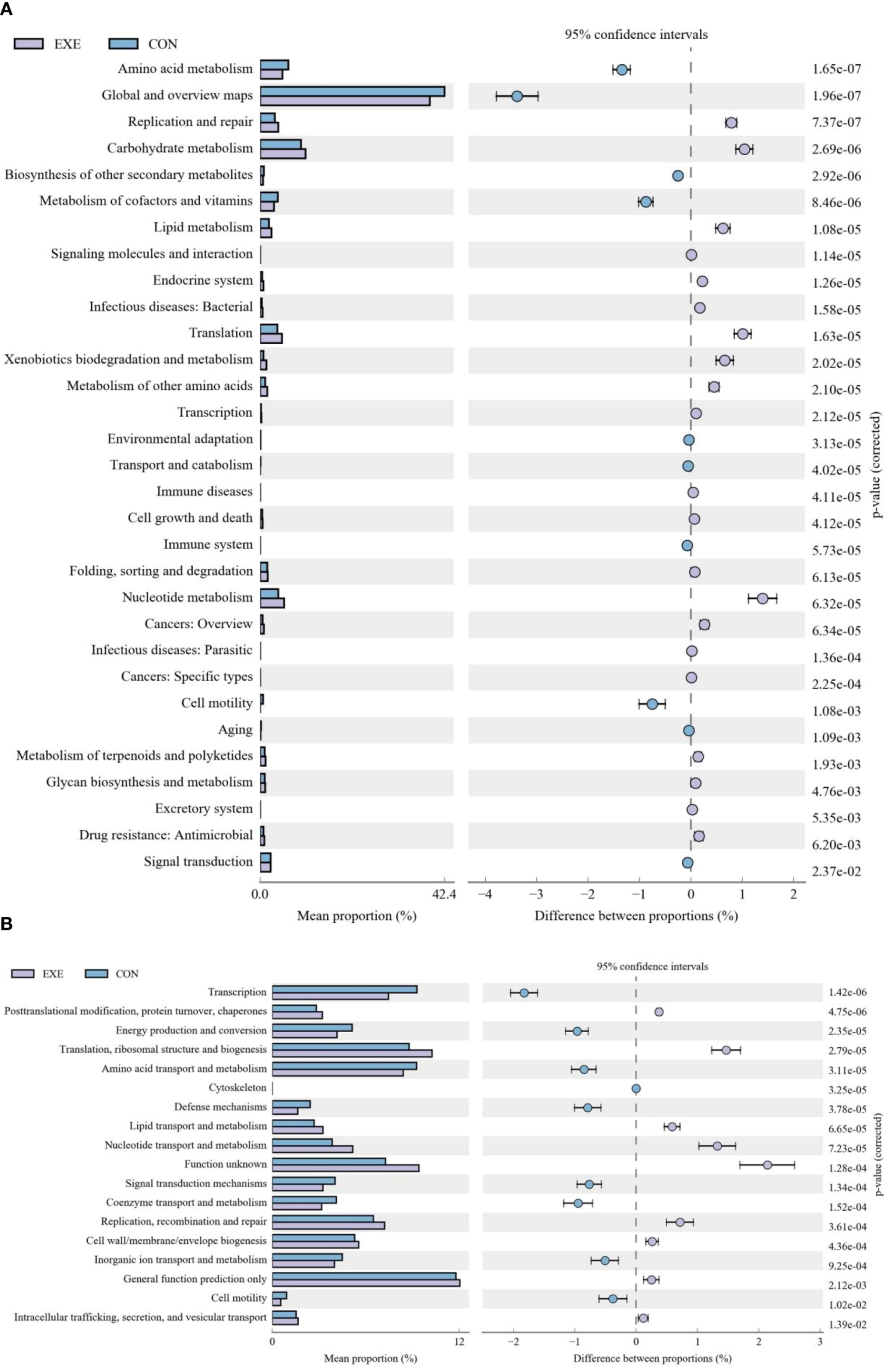
Effects of battery exposure on intestinal function in broiler chickens analyzed by KEGG **(A)** and COG **(B)**.

In the KEGG functional prediction analysis, the EXE had significantly higher relative abundances of carbohydrate metabolism, lipid metabolism, amino acid metabolism, nucleotide metabolism, metabolism of terpenoids and polyketides, xenobiotics biodegradation and metabolism, metabolism of other amino acids, glycan biosynthesis and metabolism, translation, drug resistance: Antimicrobial, transcription, replication and repair, endocrine system, signaling molecules and interaction, cell growth and death, excretory system and immune diseases, whereas metabolism of cofactors and vitamins, energy metabolism, biosynthesis of other secondary metabolites, global and overview maps, signal transduction, cell motility, folding, sorting and degradation, transport and catabolism, aging, immune system, environmental adaptation, endocrine and metabolic diseases, digestive system and neurodegenerative diseases were observed to be more abundant in the CON (**Figure 10A**). As for the COG functional prediction analysis, the relative proportions of nucleotide transport and metabolism, lipid transport and metabolism, translation, ribosomal structure and biogenesis, replication, recombination and repair, cell wall/membrane/envelope biogenesis, posttranslational modification, protein turnover, chaperones, general function prediction only, function unknown and intracellular trafficking, secretion, and vesicular transport in the EXE was significantly higher than that in the CON, whereas the relative proportions of energy production and conversion, amino acid transport and metabolism, coenzyme transport and metabolism, transcription, cell motility, inorganic ion transport and metabolism, signal transduction mechanisms, defense mechanisms and cytoskeleton was lower (**Figure 10B**).

## Discussion

As a crucial energy carrier, battery play an irreplaceable role in various sectors, including industry, agriculture, and daily life (Wang et al., 2009). However, the recycling and reuse of battery remain significant challenges that cannot be overlooked. Ineffective recycling of discarded battery may lead to the release of toxic components into the ecological environment, ultimately compromising environmental health and food safety (Gottesfeld et al., 2018). Currently, the environmental pollution and increased governance costs associated with battery waste leakage have garnered the attention of numerous countries and institutions. Furthermore, the threat posed by battery pollution to public health and livestock production has also attracted widespread concern. Several studies have indicated the detrimental effects of batteries on the health of zebrafish and mice (Liao et al., 2022; Wang et al., 2022c). Previous studies mainly focused on model animals, with limited research conducted on the effects of battery exposure on chickens. In this study, we investigated the potential toxic effects of battery leakage on broiler chickens.

The diversity index of the gut microbiota serves as a crucial indicator of gut microbiota homeostasis and is influenced by various factors (Wen et al., 2024; Chen et al., 2025b). Generally, the gut microbial diversity index correlates positively with microbial plasticity and metabolic capacity (Laitinen and Mokkala, 2019). Conversely, gut microbial diversity is recognized as a significant marker for several diseases (Li et al., 2016). For instance, a reduction in gut microbial diversity has been closely linked to the development of chronic diseases, including colitis, diabetes, and Alzheimer’s disease (Kathania et al., 2020; Cheng et al., 2023). In this study, we observed a significant decrease in the diversity and abundance of the gut microbiota in chickens exposed to battery, indicating gut microbial dysbiosis (Sun et al., 2022; Wang et al., 2022b). Notably, previous research has also revealed the adverse effects of battery exposure on the gut microbiota in mice. Numerous research suggests that gut microbial dysbiosis can adversely affect host health and intestinal function. For instance, gut microbial dysbiosis may result in increased intestinal permeability, allowing endotoxins to traverse the intestinal barrier and enter the bloodstream, resulting in systemic effects. In this study, we found significant changes in the spleen, thymus, and antioxidant-related indices in chickens following battery exposure. The spleen is a crucial lymphoid organ in the host, performing multiple functions, including immunity, blood storage, hematopoiesis, and blood filtration. Additionally, the spleen maintains blood health and the host defense capabilities by removing aging red blood cells, storing platelets, and producing immune cells (Rosado et al., 2018). The thymus serves as an important lymphatic and endocrine organ, with core functions that include T cell differentiation and maturation, immune regulation, and endocrine effects (Lewis et al., 2019; Du et al., 2020). Therefore, battery exposure may influence the immune function of chickens by impacting spleen and thymus indices. Furthermore, gut microbial dysbiosis is closely linked to the immune system. However, whether exposure to batteries can affect host immunity through alterations in gut microbioat requires further investigation.

Importantly, this research also indicated that battery exposure cause distinct changes in some functional bacteria, which may be critical for host health and intestinal homeostasis. Notably, most of these quantitatively decreased bacteria (*Coprococcus*, *Lachnospiraceae_NK4A136_group*, *Ruminococcus_torques_group*, *Lachnospiraceae_NC2004_group*, *Ruminococcus_gauvreauii_group*, *Oscillibacter*, *Alistipes*, *Intestinimonas*, *Ruminococcus*, *Roseburia*, *Romboutsia*, *Faecalibacterium*, *Bacillus*, *Lachnospiraceae_UCG_008*, *Blautia*, *Lachnospiraceae_UCG_010*, *Lachnospiraceae_UCG_006*, *Lachnospiraceae_FCS020_group*, *Acetitomaculum* and *Bifidobacterium*, etc.) in the battery exposure group were deemed as intestinal beneficial bacteria, implying that the current intestinal environment is not conducive to the survival of these bacteria. *Faecalibacterium* possesses numerous important physiological functions and plays a crucial role in gut-host interactions. Previous research indicated that *Faecalibacterium* is negatively correlated with type 2 diabetes, non-alcoholic fatty liver disease (NAFLD), and obesity (Ganesan et al., 2018). Conversely, supplementation with *Faecalibacterium* can reduce plasma endotoxin (LPS) concentrations and maintain gut microbial balance, thereby alleviating conditions such as atherosclerosis, colitis, and NAFLD (Xu et al., 2020; Safarzadeh et al., 2025). The *Lachnospiraceae* is widely distributed throughout the animal gastrointestinal tract and is essential for energy metabolism, immune regulation, and disease progression (Zhang et al., 2023a). *Ruminococcus* effectively breaks down complex polysaccharides, including resistant starch and plant cell walls, by secreting cellulases and hemicellulases, which enhances host energy utilization (Pal et al., 2021). Furthermore, abnormal levels of *Ruminococcus* have been closely associated with obesity, diabetes, and enteritis (Nie et al., 2025). Previous studies indicated that *Butyricicoccus* can ferment dietary fiber to produce butyrate via the Acetyl-CoA pathway, playing a critical role in maintaining the intestinal barrier and combating inflammation (Boesmans et al., 2018). *Christensenellaceae* has been linked to host metabolic health, participating in the degradation of complex polysaccharides and maintaining gut microbiota balance (Waters and Ley, 2019). *Flavonifractor*, a gram-negative anaerobic bacterium, exhibits anti-inflammatory properties and protects the intestinal barrier (Mikami et al., 2020). Additionally, the abundance of *Flavonifractor* is significantly reduced in patients with irritable bowel syndrome, inflammatory bowel disease, and type 2 diabetes. *Bifidobacterium*, a core beneficial gut bacterium, regulates various physiological functions, including immune homeostasis, energy metabolism, and neuroendocrine function, through microbiota-host interactions (Gavzy et al., 2023). Research indicates that *Bifidobacterium* not only preserves intestinal barrier integrity by competitively inhibiting pathogen colonization but also directly modulates host epigenetic modifications via the secretion of active substances such as exopolysaccharides (Meng et al., 2024). Furthermore, *Bifidobacterium* specifically recognizes Toll-like receptor 2 (TLR2) on dendritic cells, initiating downstream signaling pathways and significantly enhancing the secretion of the anti-inflammatory cytokine IL-10 (Akkerman et al., 2025). Recent investigations have also highlighted the potential of *Bifidobacterium* in alleviating intestinal inflammation, obesity, and diabetes (Zhang et al., 2025). Numerous studies have shown that *Bacillus* produces broad-spectrum natural antimicrobial substances that not only directly inhibit the growth of pathogenic bacteria but also disrupt their cell membrane structures (Choyam et al., 2021). Additionally, *Bacillus* can enhance host immunity by increasing macrophage phagocytosis, the secretion of anti-inflammatory factors, and immunoglobulin levels (Wang et al., 2022a). In the livestock industry, *Bacillus* is widely utilized as a new, green feed additive to maintain livestock health and promote growth performance by balancing gut microbiota, enhancing intestinal immunity, and secreting digestive enzymes (Chen et al., 2021; Yu et al., 2024). Notably, battery exposure also led to a significant reduction in some bacteria (*Bifidobacterium*, *Bacillus*, *Flavonifractor, Christensenellaceae, Acetitomaculum*, *Blautia*, *Roseburia, Intestinimonas*, *Coprococcus*, *Lachnospiraceae_NK4A136_group*, *Lachnospiraceae_NC2004_group*, *Lachnospiraceae_UCG_010*, *Lachnospiraceae_UCG_006*, *Lachnospiraceae_FCS020_group*, *Ruminococcus_torques_group, Ruminococcus_gauvreauii_group*, *Flavonifractor*, *Oscillibacter*, *Alistipes*, *Faecalibacterium* and *Butyricicoccus*) that produce SCFAs. SCFAs, recognized as pivotal signaling molecules in the interaction between gut microbiota and the host, have garnered significant attention in recent years due to their multifaceted roles in maintaining intestinal homeostasis and modulating host metabolism and immunity (Jadhav et al., 2022; Ma et al., 2022a). For instance, SCFAs can exert systemic effects on the host’s nervous, endocrine, and cardiovascular systems by mediating the activation of G protein-coupled receptors and inhibiting histone deacetylases (Lu et al., 2016; Yang et al., 2018). Furthermore, diminished levels of SCFAs can compromise the integrity of the intestinal barrier, causing endotoxemia and chronic inflammation, which may promote insulin resistance and fat accumulation, ultimately resulting in obesity and type 2 diabetes (Ma et al., 2022b). Notably, SCFAs also play a crucial role in autoimmune diseases such as rheumatoid arthritis and inflammatory bowel disease by regulating the balance between Th17 and Treg cells (Sun et al., 2017). Interestingly, previous studies have also demonstrated the effects of battery exposure on the gut microbiota of mice, which is accompanied by a significant reduction in beneficial bacteria (Liao et al., 2022). These findings highlight the negative impact of battery exposure on the host’s intestinal homeostasis.

## Conclusion

In conclusion, this study investigated the negative effects of battery exposure on gut microbiota in chickens. Results indicated that battery exposure can lead to gut microbial dysbiosis, primarily characterized by decreased microbial diversity and altered microbial composition. Furthermore, battery exposure also results in abnormalities in immune organ indices and serum biochemical markers. This study provides detailed and novel insights into the interplay between gut microbiota and host health under battery exposure conditions. Additionally, it expands the understanding of the toxic effects of battery, revealing that gut microbial dysbiosis may be significant mechanisms through which battery exerts its toxic effects. Future research should focus on the potential role of gut microbiota in monitoring poisoning and treating toxic diseases in animals.

## Data Availability

The datasets presented in this study can be found in online repositories. The names of the repository/repositories and accession number(s) can be found in the article/supplementary material.
